# Viral hepatitis micro-elimination: models of care and barriers to implementation in 5 EU/EEA prisons

**DOI:** 10.1093/eurpub/ckac129.387

**Published:** 2022-10-25

**Authors:** T Seyler, L Montanari, L Ceccarelli, E Torri, S Mazzilli, L Tavoschi

**Affiliations:** 1 Public Health Unit, EMCDDA, Lisbon, Portugal; 2 Department of Translational Research in Medicine, University of Pisa, Pisa, Italy; 3 Scuola Normale Superiore, Pisa, Italy

## Abstract

**Introduction:**

Coverage of essential prevention and control services and adequate monitoring schemes for viral hepatitis are often suboptimal in prison settings. Yet, evidence shows that targeted interventions are feasible and effective in reducing viral hepatitis burden and decreasing virus circulation among people living in prison and the community at large. To promote transferability and improvement of prison health quality in EU/EEA the European Monitoring Centre for Drugs and Drug Addiction (EMCDDA) will identify and disseminate models of care for viral hepatitis elimination in prisons.

**Methods:**

The models of care were gathered using a data collection tool that has been designed for this purpose based on the literature review and agreed with an expert advisory group. Based on the results of the data collection, a survey for healthcare staff working in 5 selected prison institutions in the EU/EEA has been developed.

**Results:**

The following models of care were collected: HCV micro-elimination in prison; transitional care for HCV treatment or HBV prevention/treatment; HCV or HBV care services tailored to women living in prison; HBV or HAV/HBV vaccination in prison settings. Harm reduction and drug treatment services in the prison are essential at all steps of the prevention and continuum of care. Among barriers identified were: engagement of people living in prison and prison governance structure, availability of infrastructural and human resources, daily prison organisation, inter-sectorial collaboration within prison and between prison and community services, training for prison staff and lack of systematic monitoring.

**Conclusions:**

Evidence of effective and acceptable interventions in prison to prevent and control viral hepatitis is essential to foster inclusion of prison setting within national elimination programmes. Intra-EU benchmarking may help promote awareness, to allocate adequate resources, monitor of impact and ultimately the achievement of the elimination goal.

